# Conflicts of interest and the role of governments in tobacco control

**DOI:** 10.18332/tid/205797

**Published:** 2025-07-23

**Authors:** Pablo Campins Bover, Laurent Huber, Laura Salgado, Chris Bostic

**Affiliations:** 1 Action on Smoking and Health, Madrid, Spain; 2 Action on Smoking and Health, Washington, United States; 3 Global Center for Good Governance in Tobacco Control, Bangkok, Thailand

**Keywords:** tobacco control, conflicts of interest, public health policy, government regulation, youth advocacy

Despite being responsible for 100 million deaths in the 20th Century and continuing to cause over 8 million deaths annually^1^, the tobacco industry continues to have substantial influence in shaping public health policies. Through strategies including lobbying, political financing, and manipulation of scientific research, it molds legislation and regulations in its favor – a fundamental conflict of interest^2^ that jeopardizes public health efforts. This guest editorial examines these conflicts of interest and the crucial role that governments must play in safeguarding public health, with particular attention to the adoption of measures that protect youth as well as adults, while ensuring compliance with international health and human rights commitments such as the WHO Framework Convention on Tobacco Control (FCTC)^3^.

## Conflicts of interest in the tobacco industry

The tobacco industry has a long history of interfering in public health policy formulation^4^. It leverages its economic power to influence legislators and regulators with the aim of weakening, delaying, or blocking policies that would reduce tobacco consumption. This conflict of interest is evident in several key practices:

1. Lobbying and political financing: Tobacco companies invest millions of dollars in lobbying and political campaign donations. This enables them to directly access lawmakers, promoting and favoring their interests over public health concerns. In many countries, politicians accept tobacco funding for campaigns^5,6^ or even move between government and tobacco industry through the revolving door^6,7^, creating a conflict of interest and undermining policy integrity.
2. Corporate social responsibility (CSR): Tobacco companies’ CSR campaigns seek to improve their public image and gain political support by portraying themselves as responsible and philanthropic actors, despite the acknowledged harm caused by their products^8^.
3. Science manipulation: A well-documented strategy of the tobacco industry involves funding research that downplays the risks of tobacco use or promotes so-called reduced-risk products such as e-cigarettes. By selectively supporting studies with favorable outcomes, the industry seeks to manufacture doubt about the harms of its products, misleading both policymakers and the public. This manipulation of scientific evidence perpetuates regulatory inertia and constitutes a profound conflict of interest^9^. Biased funding of scientific research is another common tactic. The industry sponsors studies that downplay tobacco risks or exaggerate the benefits of reduced-harm products like e-cigarettes.

## The role of governments

Governments have the responsibility to protect public health and must act firmly to counter the influence of the tobacco industry^10^. Several essential strategies should be highlighted:

1. Strict regulation and oversight: It is crucial for governments to implement and rigorously monitor regulations concerning the advertising, promotion, and sale of tobacco products, including new products like e-cigarettes. This involves effectively enforcing the WHO Framework Convention on Tobacco Control, accelerating its implementation in line with UN Sustainable Development Goal 3 and Target 3a^3^. It is essential to implement a comprehensive ban on all forms of advertising, promotion and sponsorship, including so-called ‘corporate social responsibility’ of tobacco products^11^, use tax and price measures to discourage the consumption of these harmful products, insert large health warnings on product packaging, protect all citizens from exposure to tobacco smoke, provide access to cessation programs, and hold the tobacco industry liable for the harm it causes.
2. Transparency and monitoring: It is crucial to establish transparency mechanisms for monitoring tobacco industry lobbying activities. This may include public records of meetings between government officials and industry representatives, promoting political transparency^12^.
3. Protection of public health policies: Adopting and implementing Article 5.3 of the WHO FCTC, which urges governments to protect public health policies from commercial interests of the tobacco industry^13^.
4. Promotion of healthy lifestyle: Promoting healthy alternatives to tobacco through youth education and prevention campaigns, supporting tobacco cessation programs focused on quitting smoking, highlighting the improvement in quality of life without tobacco, and preserving the health of the dependent individual’s environment. Special emphasis should be placed on reducing the risk of cancer, heart attacks, and cardiovascular diseases^14^.

## Global youth voices perspective

The Kigali Youth Declaration raised concerns about youth’s vulnerability to exposure to modifiable risk factors like tobacco and e-cigarettes, and the Global Youth Voices (GYV)^15^, a movement that represents youth coalitions and organizations from across the globe that seek to make the tobacco industry pay for the harms caused to the planet and its people, not only for those in this generation but also future generations, and adopted a Declaration in 2024^16^. This Declaration urgently calls on governments worldwide to take concrete and effective measures to protect the environment. It calls for more ambitious policies promoting a sustainable future without tobacco industry interference. Furthermore, it demands significant youth inclusion in decision-making and the implementation of clear, verifiable actions to ensure a sustainable future for all.

The GYV Declaration 2024^17^ emphasizes the need to ban cigarette filters and disposable vaping devices which should be classified as hazardous waste. This measure not only addresses a significant environmental issue but also represents an innovative public health strategy. Filters and disposable devices contribute to environmental damage^18^ and potentially perpetuate tobacco consumption among young people. Banning these products can:

1. Reduce environmental impact: By eliminating cigarette filters and disposable vaping devices, the amount of toxic waste polluting the environment is reduced^19,20^.
2. Decrease tobacco consumption: By making it harder to access disposable tobacco products, consumption among young people, who are more likely to start with easily accessible and attractive products, can be reduced. Eliminating filters may also potentially decrease tobacco use initiation and encourage cessation.

## Innovations and proactive measures

To further strengthen the efforts to reduce tobacco industry influence, several innovations and proactive measures can be considered:

1. Education and awareness: Developing youth education programs to increase awareness of tobacco dangers and industry tactics, empowering youth to make informed decisions and actively participate in public health campaigns^21^.
2. International collaboration: Promoting international collaboration to share strategies and best practices in resisting tobacco industry interference, leveraging successful experiences from different countries.
3. Responsibility and compensation: Governments must ensure that the tobacco industry assumes financial responsibility for the harm caused. Recently, at the Tenth Conference of the Parties to the WHO Framework Convention on Tobacco Control (FCTC), a decision was made to accelerate implementation of Article 19 of the FCTC, which addresses the financial responsibility and legal liability of the tobacco industry^22^. This article establishes mechanisms for the industry to assume responsibility for the damage caused by its products, including imposing taxes, penalties, and compensation mechanisms that compel the industry to cover associated social and environmental costs. This would ensure that the costs of tobacco’s adverse effects are internalized by the industry, thereby promoting social and environmental justice^23^.
4. Ban tobacco advertising, promotion and sponsorship, including those disguised as CSR initiatives: By prohibiting these practices and exposing how the tobacco industry subverts Sustainable Development Goals (SDGs) to portray itself positively in health, wellness, and environmental initiatives, governments can protect public health and ensure genuine progress toward sustainability^24^.
5. Digital solutions: One of the most impactful innovations is leveraging digital solutions. Young people play a pivotal role in harnessing digital media for tobacco control efforts. By using digital platforms, they can counter the tobacco industry’s tactics, spread awareness, and advocate for policy changes. Engaging youth in these efforts not only strengthens tobacco control but also advances the SDGs^25^.

## Conclusion

Combatting the influence of the tobacco industry requires a multifaceted approach that includes adopting strict policies, holding the industry financially accountable, and protecting public health policy formulation processes. Governments must act decisively and commit to ensuring that tobacco commercial interests do not compromise best practice policies that protect the health and well-being of current and future generations. The GYV Declaration 2024 serves as an urgent call to action and a reminder of the critical importance of prioritizing public health over commercial interests. This approach will not only protect citizen health but also contribute to a healthier and more sustainable environment for future generations.

## Data Availability

Data sharing is not applicable to this article as no new data were created.
